# Ketoacidosis at diagnosis of diabetes mellitus type 1: impact of the COVID-19 pandemic

**DOI:** 10.1590/1806-9282.20260238

**Published:** 2026-08-24

**Authors:** Denise Bousfield da Silva, Rúbia Caroline Paz Roseno da Silva, Jefferson Traebert, Marilza Leal Nascimento

**Affiliations:** 1 Universidade Federal de Santa Catarina, Department of Pediatrics – Florianópolis (SC), Brazil.; 2 Universidade do Sul de Santa Catarina, Postgraduate Program in Health Sciences – Palhoça (SC), Brazil.; 3 Universidade Federal de Santa Catarina – Florianópolis (SC), Brazil.

**Keywords:** Type 1 diabetes mellitus, Diabetic ketoacidosis, COVID-19, Child, Adolescent

## Abstract

**OBJECTIVE:**

The objective of this study was to identify the frequency and severity of diabetic ketoacidosis in the diagnosis of type 1 diabetes mellitus in the pre-, during-, and post-COVID-19 pandemic periods at a state-reference pediatric center.

**METHODS:**

Observational, cross-sectional period study, including medical records of patients diagnosed with type 1 diabetes mellitus from 2018 to 2024. The association between the diagnosis period and the presence of diabetic ketoacidosis was assessed using odds ratios with 95%CIs. The association between the diagnosis period and the need for intensive care unit admission, symptom duration, and previous incorrect diagnoses was assessed using Fisher's exact test. Temporal trends in diabetic ketoacidosis severity were assessed using the Cochran-Armitage trend test. p<0.05 were considered significant.

**RESULTS:**

Of the 184 type 1 diabetes mellitus cases, 55.4% presented with diabetic ketoacidosis at diagnosis. Patients diagnosed during the pandemic had a higher chance of developing diabetic ketoacidosis compared to the pre-pandemic period (OR 3.17; 95%CI 1.52–6.64; p=0.002) and the post-pandemic period (OR 2.90; 95%CI 1.37–6.16; p=0.005). There was no significant association between the diagnosis period and the occurrence of previous incorrect diagnoses, between the diagnosis period and the time of symptom progression, and intensive care unit admission. Regarding the severity of diabetic ketoacidosis, no significant temporal trend was identified (p>0.05).

**CONCLUSION:**

An association was observed between the pandemic period and the occurrence of diabetic ketoacidosis, without inference of causality. There are no significant differences in the proportion of severe diabetic ketoacidosis during the pandemic period.

## INTRODUCTION

Type 1 diabetes mellitus (T1DM) is an autoimmune disease in more than 90% of cases. This process is believed to be triggered by damage to beta cells from environmental factors, mainly infections, in genetically susceptible individuals^1^.

Diabetic ketoacidosis (DKA) is present in 15–70% of initial diagnoses of the disease^1,2^. It is a serious complication, responsible for a significant number of hospitalizations and 1.5–3% of deaths in this group of patients^1^.

The difficulty of accessing health services, due to fear of seeking care and mobility restrictions during the COVID-19 pandemic, impacted public health, thus worsening the clinical conditions of patients with other diseases, including T1DM1^3,4^.

In this context, the objective of this study was to evaluate the frequency and severity of DKA in the diagnosis of T1DM during the COVID-19 pandemic and compare it with the pre- and post-pandemic periods.

## METHODS

This was an observational, cross-sectional, periodic study analyzing electronic medical records of patients under 15 years of age admitted to the Joana de Gusmão Children's Hospital (HIJG) between January 2018 and June 2024, with ICD codes E10 and E10.1. Patients with DKA at the time of diagnosis of T1DM were included for analysis. Patients with incomplete data and other forms of diabetes were excluded.

This research was registered on Plataforma Brasil, CAEE 79876524.7.0000.5361, and approved by the Ethics Committee involving Human Beings (CEP) of HIJG under number 6.967.235.

This study used the definition of T1DM from the American Diabetes Association (ADA). The diagnosis and severity of DKA were established based on the venous blood gas reference values from the ADA and the International Society for Pediatric and Adolescent Diabetes (ISPAD)^2,5^.

To compare the number of admissions and the profile of these patients, the pre-pandemic period was assumed to be January 28, 2018, to March 10, 2020; the pandemic period, March 11, 2020, to April 22, 2022; and the post-pandemic period, April 23, 2022, to June 3, 2024.

COVID-19 testing was not performed on patients due to unavailability at the time.

Demographic, clinical-epidemiological, diagnostic, and evolution variables were analyzed. The age range was stratified according to Marcondes' classification^6^.

Categorical variables were described in absolute and percentage frequencies. The association between the timing of T1DM diagnosis (pre-, during-, and post-pandemic) and the presence of DKA was assessed using odds ratios (OR) with 95%CI. The association between the timing of diagnosis and the need for intensive care unit (ICU) admission, the duration of symptoms, and the occurrence of previous incorrect diagnoses was assessed using Fisher's exact test. p<0.05 were considered statistically significant.

DKA severity was categorized as mild, moderate, or severe. Comparisons between groups were performed using the chi-square test of independence. Additionally, temporal trends in DKA severity were assessed using the Cochran-Armitage trend test. To assess the risk of severe DKA, data were dichotomized (severe versus non-severe), and the OR with the respective 95%CIs was calculated. Fisher's exact test was used for pairwise comparisons when appropriate. A two-tailed p<0.05 was considered statistically significant.

## RESULTS

A total of 184 medical records of patients diagnosed with T1DM admitted to HIJG were analyzed, of which 102 (55.4%) presented with DKA at diagnosis. Of the 102 patients with DKA, 28 (27.5%) occurred in the pre-pandemic period, 47 (46.1%) during, and 27 (26.4%) post-pandemic. Of the 82 cases without DKA, 34 (41.5%) occurred in the pre-pandemic period, 18 (21.9%) during, and 30 (36.6%) post-pandemic.

Patients diagnosed during the pandemic had a higher chance of DKA compared to the pre-pandemic period (OR 3.17; 95%CI 1.52–6.64; p=0.002) and the post-pandemic period (OR 2.90; 95%CI 1.37–6.16; p=0.005).

According to sociodemographic characteristics, DKA was more frequent (Table 1) in males (56.9%), schoolchildren (41.2%), and adolescents (32.3%). Throughout the study periods, variations in distribution by age group were observed, without statistically significant differences.

**Table 1:** Sociodemographic characteristics of pediatric patients with diabetic ketoacidosis at diagnosis, according to pre-pandemic, pandemic, and post-pandemic periods (HIJG, Florianópolis, 2018–2024).

Variable	Category	Total (n=102)*	Pre (n=28)**	During (n=47)**	Post (n=27)**
Sex	Female	44 (43.1%)	13 (46.4%)	19 (40.4%)	12 (44.4%)
	Male	58 (56.9%)	15 (53.6%)	28 (59.6%)	15 (55.6%)
Age group	Infant	5 (4.9%)	4 (14.3%)	1 (2.1%)	0 (0%)
	Preschool	22 (21.6%)	5 (17.9%)	10 (21.3%)	7 (25.9%)
	School age	42 (41.2%)	10 (35.7%)	17 (36.2%)	15 (55.6%)
	Adolescent	33 (32.3%)	9 (32.1%)	19 (40.4%)	5 (18.5%)

* Percentage relative to the overall total (n=102).

** Percentage relative to the total of each period.

The classic signs and symptoms of DKA were present in 98% of cases in all periods. Most patients with DKA had a symptom duration of less than 1 month (82.3%), regardless of the diagnosis period (Table 2). There was no statistically significant association between the diagnosis period and the symptom duration (Fisher's exact test, p≈0.78).

**Table 2:** Clinical and outcome data at diagnosis of patients with diabetic ketoacidosis at diagnosis of type 1 diabetes mellitus in the pre-pandemic, pandemic, and post-pandemic periods (HIJG, Florianópolis, 2018–2024).

Variable		Total (n=102)		Pre (n=28)		During (n=47)		Post (n=27)	
		n	%**	n	%*	n	%*	n	%*
Symptom duration									
	<1 month	84	82.3	23	82.1	37	78.7	24	88.9
	1–3 months	11	10.8	4	14.3	6	12.8	1	3.7
	>3 months	5	4.9	1	3.6	3	6.4	1	3.7
	Unknown	2	2.0	0	0	1	2.1	1	3.7
Prior diagnoses									
	Yes	20	19.6	8	28.6	9	19.1	3	11.1
	No	82	80.4	20	71.4	38	80.9	0	88.9
ICU admission									
	Yes	35	34.3	8	28.6	19	40.4	8	29.6
	No	67	65.7	20	71.4	28	59.6	19	70.4

* Percentage relative to the total for the period.

** Percentage relative to the overall total (n=102). ICU: intensive care unit.

Regarding previous incorrect diagnoses (Table 2), 19.6% of patients with DKA had received another diagnosis before confirming T1DM. This proportion was higher in the pre-pandemic period (28.6%) compared to the periods during (19.1%) and post-pandemic (11.1%); however, there was no statistically significant association between the period of diagnosis and the occurrence of previous incorrect diagnoses (Fisher's exact test, p≈0.24).

ICU admission was required in 35 patients with DKA (34.3%). The highest proportion occurred during the pandemic (40.4%), with no statistically significant association between the period of diagnosis and the need for ICU admission (Fisher's exact test, p≈0.48).

Severe DKA was observed in 50% of cases in the pre-pandemic period, 53.2% during the pandemic, and 44.5% in the post-pandemic period (Table 3). No statistically significant differences were observed in the distribution of DKA severity across the study periods (chi-square test, p>0.05). Similarly, no significant temporal trend was identified using the Cochran-Armitage test (p>0.05). When severity was dichotomized (severe versus non-severe), the probability of severe DKA was slightly higher during the pandemic period compared to the pre-pandemic period (OR 1.14) and lower in the post-pandemic period (OR 0.80), although these differences were not statistically significant (Fisher's exact test, p>0.05).

**Table 3:** Severity of diabetic ketoacidosis at diagnosis of type 1 diabetes mellitus, according to pre-pandemic, pandemic, and post-pandemic periods (HIJG, Florianópolis, 2018–2024).

Severity of DKA	Pre (n=28) n (%)	During (n=47) n (%)	Post (n=27) n (%)
Mild	5 (17.9%)	11 (23.4%)	5 (18.5%)
Moderate	9 (32.1%)	11 (23.4%)	10 (37.0%)
Severe	14 (50.0%)	25 (53.2%)	12 (44.5%)

DKA: diabetic ketoacidosis.

All patients survived.

## DISCUSSION

DKA represents an acute and potentially fatal complication of T1DM and may be present at the time of diagnosis^1^. Early recognition of the signs and symptoms of T1DM is essential to prevent DKA, a condition associated with higher complication rates and a worse long-term prognosis^7^. Thus, it becomes relevant to investigate how events of global magnitude can influence the presentation patterns of this condition in pediatric populations^7^.

Ho et al.^8^ identified that the number of children with newly diagnosed T1DM was similar during the pandemic compared to the previous year, but the frequency of DKA increased from 45.6 to 68.2% (p<0.001), indicating that the pandemic did not modify the incidence of the disease, but altered its severity.

A meta-analysis^9^ including 124,597 children demonstrated a significant increase in the risk of DKA in patients with T1DM diagnosed during the pandemic (p<0.01).

In the present study, a balanced distribution of T1DM cases was observed across the three periods; however, patients diagnosed during the pandemic presented a higher risk of DKA compared to the pre- and post-pandemic periods (OR 2.90, 95%CI 1.37–6.16), although without causal inference. The COVID-19 pandemic, however, did not result in a sustained worsening of DKA severity (Table 3) at the time of diagnosis. The fluctuations observed across the periods may reflect random variations or transient changes in access to healthcare services and referral patterns, not reflecting a true alteration in disease presentation.

When dichotomizing the severity of DKA, a small increase in the probability of severe presentation was observed during the pandemic period, followed by a reduction in the post-pandemic period. However, these findings were not statistically significant, reinforcing the overall stability of clinical severity over time. These results suggest that, despite concerns about late diagnosis during the pandemic, the severity of metabolic decompensation at clinical presentation remained relatively stable in this study.

The clinical picture of DKA begins with classic signs and symptoms, in addition to nausea, abdominal pain, and vomiting^1,2,5^. In this case, the classic signs/symptoms were the most frequent, regardless of the period analyzed.

Regarding the time of presentation of signs/symptoms, a multicenter study conducted by Alaqeel et al.^10^, in children with recently diagnosed T1DM, demonstrated higher levels of glycated hemoglobin during the pandemic (p<0.001). A higher frequency of DKA at diagnosis was also observed (p<0.001), a result attributed by the authors to delays in access to the health system in the context of overburdened services^11^.

In this study, however, it was observed that 82.3% of cases (Table 2) reported onset of signs/symptoms less than a month prior, with no significant difference between the three periods, indicating that the delay in diagnosis, by itself, does not fully explain the variability observed in clinical severity. Additionally, as this is an observational, cross-sectional period study, this aspect may reflect methodological limitations, including difficulties for caregivers in determining the exact time of symptom onset.

The conditions most commonly confused with DKA include infectious, respiratory, and gastrointestinal diseases^12,13^, possibly due to the overlap between the initial signs and symptoms of T1DM and those of other prevalent childhood illnesses.

In this study, similar to that of Muñoz et al.^13^, it was shown that 19.6% of patients (Table 2) had received other diagnoses previously, highlighting the importance of continuing medical education for the early recognition of T1DM. Despite concerns about late diagnosis during the pandemic, the results of the present study suggest that the severity of metabolic decompensation at clinical presentation remained relatively stable.

Regarding the need for ICU admission, a study conducted in Seattle^14^ identified that the proportion of patients with DKA who went to the ICU increased from 34.2% in the pre-pandemic period to 54.6% during the pandemic.

In the present research, although the highest proportion of ICU admissions occurred during the pandemic (Table 2), there was no significant association between the time of diagnosis and the need for ICU. This finding suggests that, although the pandemic impacted the risk of DKA, the severity of presentations requiring intensive support remained relatively stable. Individual factors, such as age, intensity of metabolic decompensation, and comorbidities, likely exerted a greater influence on the need for ICU than the epidemiological period itself.

In this study, although a slight increase in the proportion of severe cases was observed during the pandemic period (Table 3), this variation was not statistically significant. The small fluctuations observed over the periods may reflect random variations or transient changes in access to health services and referral patterns, rather than a true change in disease presentation. When dichotomizing the severity of DKA, a small increase in the probability of severe presentation was observed during the pandemic period, followed by a reduction in the post-pandemic period. However, these findings were not statistically significant, reinforcing the overall stability of clinical severity over time.

All patients survived, which possibly reflects the quality of care provided at this referral center.

This study presented limitations that should be acknowledged, such as potential confounding factors, including the presence of COVID-19 infection in children with KDA and among their household contacts, as well as the lack of objective measures to assess parental hesitancy in seeking medical care during the pandemic.

Active data retrieval was performed in this study to minimize information bias and residual bias. However, there were limitations inherent to the retrospective observational design, such as possible inaccuracies in medical records, missing data, and sample size, which may have limited the statistical power of the analyses.

Selection bias is also a relevant consideration, since this study was conducted in a tertiary referral center, which may have received a higher proportion of severe cases.

Additionally, the use of aggregated data limited the ability to perform multivariate analyses and adjust for potential confounding factors, thus restricting causal inference.

## Data Availability

The datasets generated and/or analyzed during the current study are available from the corresponding author upon reasonable request.
